# The Impact of Left Ventricular Diastolic Dysfunction on Respiratory Adverse Events in Cardiac Surgery Patients—An Observational Prospective Single-Center Study

**DOI:** 10.3390/jcm12154960

**Published:** 2023-07-28

**Authors:** Marta Braksator, Magdalena Jachymek, Karina Witkiewicz, Wojciech Witkiewicz, Małgorzata Peregud-Pogorzelska, Katarzyna Kotfis, Jarosław Kaźmierczak, Mirosław Brykczyński

**Affiliations:** 1 Department of Cardiology, Pomeranian Medical University, 70-111 Szczecin, Poland; mtuniab@gmail.com (M.B.); magdajachymek@gmail.com (M.J.); witkiewiczwojciech@gmail.com (W.W.); m1peregud@gmail.com (M.P.-P.); jar.kazmierczak@o2.pl (J.K.); 2 Department of Pulmonology, Pomeranian Medical University, 70-204 Szczecin, Poland; karina.witkiewicz@gmail.com; 3 Department of Anaesthesiology, Intensive Therapy and Acute Intoxications, Pomeranian Medical University, 70-111 Szczecin, Poland; 4 Department of Cardiac Surgery, University of Zielona Góra, 65-417 Zielona Góra, Poland; mirobryk@gmail.com

**Keywords:** LV diastolic dysfunction, postoperative respiratory adverse events, pneumonia

## Abstract

Background: Left ventricular diastolic dysfunction (LV DD) is the most dominant cause of heart failure with preserved ejection fraction (HFpEF) worldwide. This pathological condition may contribute to postcapillary pulmonary hypertension (pcPH) development. Hypoxemia, often observed in pcPH, may significantly negatively impact the course of hospitalization in patients after cardiac surgery. The aim of our study was to investigate the impact of LV DD on the frequency of postoperative respiratory adverse events (RAE) in patients undergoing Coronary Artery Bypass Grafting (CABG). Methods: The left ventricular (LV) diastolic function was assessed in 56 consecutive patients admitted for CABG. We investigated the relationship between LV DD and postoperative respiratory adverse events (RAE) in groups with normal LV diastolic function and LV DD stage I, II, and III. Results: Left ventricular diastolic dysfunction stage I was observed in 11 patients (19.6%) and LV DD stage II or III in 19 patients (33.9%). Arterial blood partial pressure of oxygen (PaO2) to the fraction of inspired oxygen (FiO2) index during postoperative mechanical ventilation was significantly lower in LV DD stage II or III than in the group with normal LV diastolic function. Patients with DD stage II or III had a higher occurrence of postoperative pneumonia than the group with normal LV diastolic function. Conclusions: Left ventricular diastolic dysfunction is widespread in cardiac surgery patients and is an independent risk factor for lower minimal PaO2/FiO2 index during mechanical ventilation and higher occurrence of pneumonia.

## 1. Introduction

Left ventricular diastolic dysfunction is the most common form of heart failure with a preserved left ventricular ejection fraction (HfpEF) phenotype [1]. HfpEF is currently the dominant form of heart failure requiring hospitalization [2]. Diastolic dysfunction (DD) is a common pathology in patients undergoing cardiac surgery (CS) [3]. In the pathophysiology of DD, we can distinguish three stages. At the beginning (DD stage I), impaired relaxation of the LV myocardium is observed, which is reflected in the decrease in early diastolic mitral annular velocity (e’) in transthoracic echocardiography (TTE). In time, when LV loses its restoring forces, LV stiffness increases, and its filling pressure heightens, which causes the elevation of left ventricular end-diastolic pressure (LVEDP). The elevated pressures are passively transmitted into the left atrium (LA). In TTE, we observe low e’ velocity and high early (E) and late (A) mitral inflow velocities. The E/A ratio remains within the scope of the standard, but both velocities are elevated—we can thus recognize DD stage II (the so-called pseudonormalization). In time, the left atrium enlarges, and its pressure increases. This state is characteristic of DD stage III and can promote congestion of the pulmonary veins and postcapillary Pulmonary Hypertension (pcPH) development [4]. It is reflected in the elevated left atrial volume index (LAVI) and tricuspid valve regurgitation maximal velocity (TRVmax) in TTE. The PH is an independent risk factor of mortality after cardiac surgery [5]. Hypoxemia and hypocapnia, often observed in PH [6], may be deepened during cardiopulmonary bypass (CPB). Most studies assessed the impact of LV DD on the length of mechanical ventilation and duration of hospitalization in CS patients. However, most of them do not give the reasons for prolonged mechanical ventilation. LV DD’s impact on pneumonia, which is a severe complication in CS, is still unexplored [3,7]. Our study aimed to investigate the LV DD’s impact on the occurrence of postoperative RAEs, defined as hypoxemia and pneumonia, in patients undergoing CABG with CPB. 

## 2. Materials and Methods 

The procedures of this study followed good clinical practice (GCP). The study protocol was approved by The Bioethics Committee of the Pomeranian Medical University in Szczecin 10.06.2019 (KB-0012/172/06/19). Eligible patients received oral and written information at least 24 h before the operation. The study was performed between November 2019 and September 2021 in the Cardiac Surgery Department of Pomeranian Medical University in Szczecin, Poland. 

### 2.1. Inclusion and Exclusion Criteria of the Study 

The inclusion criteria for the study were: age over 18 years; chronic coronary artery disease (CAD), qualified for coronary artery bypass grafting with the use of cardiopulmonary bypass; elective surgery; and left ventricular ejection fraction (LVEF) ≥ 40%. The following were exclusion criteria: pulmonary diseases with a severe or moderate restrictive or obstructive disorder; mitral, tricuspid, aortic, or pulmonic valve insufficiency or stenosis qualified for operation according to 2021 ESC/EACTS Guidelines for the management of valvular heart disease [8]; non-elective surgery; LVEF < 40%; infective endocarditis; hypertrophic cardiomyopathy; atrial fibrillation or post-pacemaker/cardioverter defibrillator implantation status; pulmonary arterial hypertension diagnosed preoperatively according to right heart catheterization and perioperative myocardial infarction (MI type 5), defined according to the fourth universal definition of myocardial infarction [9]. Initially, sixty-two patients were included in the study. Five patients were excluded postoperatively due to hemorrhagic complications requiring surgical revision, and one patient was excluded due to post-CABG myocardial infarction requiring emergency angioplasty.

### 2.2. Echocardiographic Measurements 

A certified echocardiographer performed all studies 24 h preoperatively in the Echocardiography Workroom of the Cardiac Surgery Clinic of the Pomeranian Medical University (Philips EpiqCvx; Software Version: 3.0.3; Philips Ultrasound Inc., Bothell, WA, USA).

The left ventricular ejection fraction (LVEF) was estimated using Simpson’s method. Left ventricular diastolic dysfunction and its stage were defined according to the American Society of Echocardiography and the European Association of Cardiovascular Imaging 2016 Guidelines [10]. Initially, we assessed the LVEF. The diagnosis pathway depended on the left ventricular ejection fraction (mildly reduced LVEF: greater than or equal to 40% and less than 50%; or preserved LVEF: greater than or equal to 50%). The scheme of the LV diastolic dysfunction assessment is shown in Appendix A.

### 2.3. Perioperative Management and Surgery Procedures

Every patient qualified for the study received a single dose of oral benzodiazepine one hour before the operation. CABG with the use of cardiopulmonary bypass (CPB) was performed in every patient. 

#### 2.3.1. General Anesthesia Procedures

Every patient underwent general anesthesia with a single intravenous bolus of opioids and etomidate during induction of the procedure and with use of pancuronium as a myorelaxant agent. The anesthesia was maintained with an inhaled agent (Sevoflurane). No patients received inhaled nitric oxide.

#### 2.3.2. Postoperative Management and Measurements

In the Intensive Care Unit, postoperative care was provided according to the scheme of the Cardiac Surgery Clinic of Pomeranian Medical University. The PaO2/FiO2 index (Horowitz Index) was measured as a ratio of partial pressure of oxygen in arterial blood (PaO2) in millimeters of mercury and the fraction of oxygen in the inhaled air (FiO2). Postoperative analgesia consisted of intravenous paracetamol every six hours and an intravenous infusion of morphine. The therapy was adequate. We did not observe any cases of postoperative pain requiring additional drugs or regional anesthesia. Pneumonia was defined according to the American Thoracic Society guidelines as the presence of typical changes in chest radiography with at least two of the following: fever, leukocytosis (white blood cell WBC > 12 G/L), or leukopenia (WBC < 4 G/L), or expectoration of pus sputum [11]. 

Acute Respiratory Distress Syndrome (ARDS) and Transfusion-related Acute Lung Injury (TRALI) were defined according to the American-European Consensus Conference Definition and The National Heart Lung and Blood Institute (NHLBI) Working Group, respectively [12,13]. 

In case of a pneumonia diagnosis, the specimen for culture and sensitivity was collected and empirical antibiotic therapy was initiated as per protocol developed in the Cardiac Surgery Department of Pomeranian Medical University in Szczecin with the use of third-generation cephalosporin (Ceftriaxone). 

### 2.4. Endpoints

The primary endpoints in this study were the minimal and maximal arterial blood partial pressure of oxygen (PaO2), minimal and maximal ratio of partial pressure of oxygen and the fraction of oxygen in the inhaled air (PaO2/FiO2 index), pneumonia, and length of mechanical ventilation. Secondary endpoints were ARDS occurrence, TRALI occurrence, length of the ICU stay, hospitalization time, and in-hospital mortality.

### 2.5. Statistical Analysis

All analyses were performed using Statistica 13 (StatSoft, Inc., Tulsa, OK, USA) software. The continuous variables are presented as mean with standard deviation (SD) or median with interquartile range. The categorical variables are presented as numbers and a percentage. We used the Welch test, Kruskall–Wallis test, or ANOVA with the Tuckey post hoc test to test for statistical significance, depending on the distribution and variance homogeneity. Categorical data were compared using the Chi2 test. The relationship between the analyzed parameters was evaluated using multivariate regression model analysis or logistic regression. The variables were chosen with a backward stepwise elimination method. We assumed *p*-value ≤ 0.05 as significant.

## 3. Results

According to echocardiography, we divided patients into three groups: normal diastolic function (ND), DDI, and DD II or III.

### 3.1. Preoperative Characteristics of the Patients

Table 1 presents the demographic data and the type of operation.

### 3.2. Occurrence of Diastolic Dysfunction and Echocardiographic Indicators of DD

Thirty patients (53.57%) had LV DD. DD stage I was observed in 11 patients (19.6%) and LV DD stage II or III in 19 patients (33.9%).

We observed significantly lower average e’ velocity in the group with diastolic dysfunction than in the group with normal LV diastolic function. LAVI was significantly higher in the LV DD stage II or III group than in groups with DD stage I and normal diastolic function. We observed a nonsignificant change between pre- and postoperative echocardiographic parameters. All the preoperative parameters defining LV diastolic and systolic functions of the left ventricle are presented in Table 2.

### 3.3. Cardiopulmonary Bypass Parameters

Time of perfusion, aortic cross-clamp, and reperfusion did not differ significantly between the three groups of patients—the results are shown in Table 3.

### 3.4. Postoperative Data

There was no difference in the rate of postoperative blood, fresh frozen plasma, and platelet transfusion between the three groups of patients.

Norepinephrine was administered in nine patients (34.61%) from the ND group, in three patients from the DDI group (27.27%), and in six patients from the DD stage II or III group (31.58%). We did not observe any need for inotropic support 24 h after the operation. There was no need for the use of intra-aortic balloon counterpulsation during the perioperative period in the whole group of patients.

Seven patients had postoperative atrial fibrillation (26.92%) from the ND group, three patients from the DDI group (27.27%), and five patients from the DD stage II or III group (26.32%)—see Table 4. We did not observe any cases of acute kidney injury requiring renal replacement therapy.

### 3.5. Primary and Secondary Endpoints

#### 3.5.1. Primary Endpoints

All the primary endpoints and potential complications are shown in Table 5.

Postoperative pneumonia was diagnosed in seven patients from the group with LV DD stage II or III and four with DD stage I or ND (38.89% vs. 10.81%; *p* = 0.027, OR 5.25 (95% CI 1.22–21.4). LV diastolic dysfunction remains significant even after adding minimal PaO2 (*p* = 0.011, OR 9.33, 95% CI 1.67–51.86) or BMI (*p* = 0.015, OR 7.27, 95% CI 1.47–35.89) to the model. PaO2 and BMI alone had no impact on pneumonia. These results should be interpreted with caution due to the small sample size. The relationship between LV diastolic dysfunction and pneumonia should be confirmed in further studies.

Pneumonia significantly prolonged the length of mechanical ventilation, ICU, and in-hospital stay—the results are presented in Table 6.

Multiple regression was used to test if diastolic dysfunction, LVEF, sex, age comorbidities (EUROSCORE II), and BMI significantly predicted minimal PaO2/FiO2 during intubation. The fitted regression model was min PaO2/FiO2 = 101.81 − 24.33 (BMI > 30) − 31.17 (DD II/III) + 2.68 × VEF (R2 corrected 0.3, *p* < 0.001). A multivariate regression model was used to test which of the DD parameters (RA area>18, e’, E/e’, LAVI, TR V max) the best predicted minimal PaO2/FiO2 during intubation. LAVI significantly predicted PaO2/FiO2 (β = −4.77, *p* < 0.001, R2 corrected 0.2).

#### 3.5.2. Secondary Endpoints

We did not diagnose any cases of ARDS or TRALI. We did not observe any in-hospital mortality cases. All the secondary endpoints are shown in Table 7.

## 4. Discussion

Left Ventricular Diastolic Dysfunction—LV DD—is frequent in cardiac surgery patients. In a study published in 2018, LV DD stage II or III occurred in 42% of patients qualified for CABG and/or aortic valve replacement [3]. We diagnosed left ventricular diastolic dysfunction in 30 patients, almost 54% of our study participants. Nineteen patients (almost 34% of participants) had LV DD stage II or III.

Pneumonia is a serious complication that determines the postoperative outcomes of cardiac surgery and increases the therapy costs. We demonstrated that pneumonia prolonged mechanical ventilation, ICU stay, and the duration of hospitalization.

The risk factors for postoperative pneumonia in CS have been investigated in many studies, but most of them included only the predictors of LV diastolic dysfunction, such as arterial hypertension and diabetes mellitus—the correlation between LV DD and postoperative pneumonia has not been proven [14,15,16,17,18].

We have proved that left ventricular diastolic dysfunction is an independent risk factor for postoperative pneumonia. Although the relationship is significant, this phenomenon should be confirmed in further studies due to a relatively small sample size.

Hypoxemia is a serious pathological condition which is associated with increased morbidity and mortality in patients after surgical procedures [19]. The arterial blood partial pressure of oxygen (PaO2) during mechanical ventilation is affected by the fraction of inspired oxygen (FiO2); consequently, in clinical practice, the PaO2/FiO2 ratio is an approved tool used to evaluate lung injury. The PaO2/FiO2 ratio is important in assessing patients with acute respiratory distress syndrome (ARDS) and correlates with mortality in ICU patients [20]. Some studies demonstrated that a low PaO2/FiO2 ratio, measured 3 h after surgery, correlates with mortality and ICU stay length in cardiac surgery patients [21]. Even though we did not find any difference in a PaO2/FiO2 ratio when comparing patients with and without postoperative pneumonia, we decided to examine the impact of LV DD on the PaO2/FiO2 index as a potentially important pneumonia risk factor in CS.

We proved that LV diastolic dysfunction significantly predicts the PaO2/FiO2 level during postoperative mechanical ventilation. This is an important observation, which reflects the pathophysiology of the disease, when elevated LV filling pressure provokes LA pressure elevation and pulmonary congestion with gasometrical abnormalities, such as hypoxemia. However, the predictive value of the PaO2/FiO2 index in cardiac surgery patients requires further research.

The correlation between echocardiographic indicators of LV DD and respiratory adverse events in cardiac surgery is a very interesting observation.

The study published in 2011 by Jun et al. proved that the E/e’ ratio is an independent risk factor for morbidity after off-pump CABG [22]. In an article published in 2005, Hedman et al. showed that mitral inflow patterns and pulmonary venous flow are stronger predictors of postoperative adverse events even than EuroSCORE in CS patients [23].

Although the correlation between LV diastolic dysfunction indicators and increased morbidity in CS has already been described, independent risk factors for respiratory adverse events remain unknown [23,24].

We proved that LAVI significantly predicted PaO2/FiO2 during mechanical ventilation, along with BMI and LVEF. LAVI is a direct echocardiographic indicator of LV DD with elevated filling pressures (diastolic dysfunction stages II and III). However, its predictive value may be affected by many pathological heart conditions, such as mitral valve stenosis or atrial fibrillation [25]. Most of the conditions, which might impact LAVI were the exclusion criteria of our study. The fact that increased LAVI is a predictor of lower PaO2 confirms that elevated LV filling pressure promotes pulmonary congestion and postcapillary pulmonary hypertension development, which is reflected in gasometric abnormalities.

LAVI and TR Vmax were significantly higher in patients with LV DD stage II and III, and average e’ was abnormal in all the patients with LV diastolic dysfunction, irrespective of its stage. This confirms the reports from some studies that e’ is the surrogate of left ventricular early filling and decreases in the early stages of LV DD when the other parameters are within the normal range [25].

A study from 2005 showed that the tissue Doppler early diastolic mitral annular velocity increased three months after coronary artery bypass grafting [23]. In our study, echocardiographic parameters of LV diastolic function did not change significantly after surgery—the reason might be the short follow-up period, limited by the SCoV-2 pandemic. Population-based studies demonstrated that LV DD predisposing factors—arterial hypertension, diabetes, and obesity—are also risk factors for coronary artery disease [26,27]. Age is another risk factor for LV DD development [28].

In our study, patients with LV diastolic dysfunction stage II and III were older. We also observed the domination of the female sex in this group, but the differences were not statistically significant. We did not observe differences in arterial hypertension, diabetes, or obesity between the three groups according to the diastolic dysfunction stage. This may result from the strictly defined inclusion and exclusion criteria and the fact that no participants were over 75 years old, which made this sample homogenous.

Interestingly, the EuroScore II result differed between the three groups. EuroScore is an established preoperative risk assessment system in cardiac surgery [29]. The score consists of many variables, such as age and diabetes mellitus, but also procedural factors (for example, urgency of operation). Most patient- and surgery-related characteristics were comparable in our three groups, but one—sPAP—was significantly different. In healthy individuals, sPAP reflects the Right Ventricular Systolic Pressure—RVSP. Increased RVSP and sPAP are characteristic of PH. In the first part of our study, published in 2022, we observed high or moderate echocardiographic probability of PH in 51.7% (n = 29) of our patients, from which 19 presented diastolic dysfunction stage II or III [30]. The impact of diastolic dysfunction on echocardiographic signs of elevated RV pressure and sPAP explains the differences in EuroScore II in our study.

In the group with normal LV diastolic function and with DD stage II or III, the average LVEF did not differ significantly (median 65 vs. 60.1%). However, LVEF in the DDI group (median 45%) was significantly lower than in the other groups. It is a fact that LV diastolic dysfunction is a pathological state which precedes development of systolic abnormalities. We did not observe any LV ejection fraction disorders in the group with normal diastolic function. The lower average LVEF in DD stage I group than DD stage II and III group may result from the higher number of patients with mildly reduced LVEF, in which indicators of diastolic dysfunction stage I (for example, e’ velocity lower than 50 cm/s or E/e’ index higher than 14, without other echocardiographic signs of DD such as LAVI over 34 mL/m^2^ or high TRVmax) were observed. Moreover, the study design with LVEF < 40% as an exclusion criterion affected the distribution of LV ejection fraction in three groups of participants.

Postoperative pain related to sternotomy requires high doses of opioids and may facilitate the development of pneumonia. A prospective study published in 2023 proved that ultrasound-guided parasternal block provides a reduction of opioid doses required for optimal pain relief and shortening hospitalization time [31]. According to a protocol established in our Cardiac Surgery Department, postoperative pain management consists of intravenous morphine infusion and boluses of paracetamol. No patient required additional pain relief therapy. We did not observe any impact of post-operative pain on RAE in our study, but it may be due to its relatively small sample size.

### Study Limitations

Our study comes with some limitations. The sample size is relatively small despite the prospective character of the study. The COVID-19 pandemic has influenced the number of operations performed in the center and follow-up possibilities.

Additional data regarding mechanical ventilation may be required, such as tidal volume; however, we included important information, such as maximal and minimal positive end-expiratory pressure and mode of mechanical ventilation.

## 5. Conclusions

Left ventricular DD is prevalent in patients qualified for CABG. In patients with LV diastolic dysfunction, we proved prolonged postoperative mechanical ventilation and lower minimal and maximal PaO2/FiO2 indices level than in people with correct LV diastolic function. Moreover, LV diastolic dysfunction is an independent risk factor for postoperative pneumonia. Left ventricular diastolic function is a significant factor of postoperative adverse events in CS, but multi-center extensive studies should be created to precisely assess its relevance.

## Figures and Tables

**Table 1:** Preoperative characteristics of the patients and type of operation.

	ND	DD I	DD II or III	*p*
n	26	11	19	
Age	66.5 (63–72)	65 (64–75)	71 (67–72)	NS
Sex M, n (%)	22 (84.62%)	10 (90.9%)	13 (68.42%)	NS
BMI	29 (28–31)	29 (28–31)	29 (28–31)	NS
EuroScore II	0.74 (0.67–0.91)	1.19 (0.87–1.48)	1.24 (1.98–2.03)	**<0.001 (a,b)**
DM, n (%)	10 (38.46)	7 (63.64%)	7 (36.84)	NS
HA, n (%)	22 (84.6)	8 (72.73)	122 (78.9)	NS
Smoking, n (%)	10 (38.46)	4 (36.36)	7 (36.84)	NS
COPD, n (%)	1 (3.85)	1 (9.09)	1 (5.26)	NS
CKD stages 3, 4 or 5	0	0	0	NA
Stroke, n (%)	1 (3.85)	0	0	NS
CABG × 2, n (%)	12 (46.15)	6 (54.54)	10 (52.63)	NS
CABG × 3, n (%)	7 (26.92)	3 (27.27)	6 (31.58)	NS
CABG × 4, n (%)	6 (23.08)	2 (18.18)	3 (15.79)	NS
CABG × 5, n (%)	1 (3.85)	0	0	NS
LIMA + RIMA, n (%)	4 (15.38)	1 (9.09)	2 (10.53)	NS

Legend: BMI—Body Mass Index, DM—Diabetes Mellitus, HA—Arterial Hypertension, COPD—chronic obstructive pulmonary disease, CKD—Chronic Kidney Disease, CABG—Coronary Artery Bypass Grafting, LIMA—left internal mammary artery, RIMA—right internal mammary artery, NA—not applicable, NS—not significant, a—the significant difference between ND and DD I group, b—the significant difference between ND and DD II or III group

**Table 2:** Preoperative echocardiographic parameters of LV systolic and diastolic function.

	ND	DD I	DD II or III	*p*
LVEF %	65 (62–70)	45.5 (42–48)	60.1 (48–67.2)	<0.001 (a,c)
E/e’	9.1 (8.32–10.6)	10.3 (8–14.1)	15.22 (10.6–15.5)	<0.001 (b)
E/A	0.96 (0.6–1.7)	0.98 (0.4–1.26)	0.97 (0.69—2.27)	NS
average e’ cm/s	8.74 (±1.94)	6.65 (±1.46)	6.64 (±1.67)	<0.001 (a,b)
LAVI (mL/m^2^)	27 (23.6–29.5)	30.2 (28–34.8)	41.55 (39.6–46.4)	<0.001 (b,c)
TR Vmax (m/s)	1.5 (1.2–2.9)	1.7 (1.4–2.9)	2.75 (2.6–2.9)	<0.001 (b,c)
sPAP	21 (13–44)	32.1 (13—48)	46.5 (35—51)	<0.001 (a,b)

Legend: LVEF—Left Ventricular Ejection Fraction, E—Early transmitral pulsed—wave Doppler flow, A—Atrial transmitral pulse—wave Doppler flow, e’—early mitral annular velocity, LAVI—Left atrial volume index, TR Vmax—tricuspid valve regurgitation maximal velocity, sPAP—systolic pulmonary artery pressure, NS—not significant, a—the significant difference between ND and DD I group, b—the significant difference between ND and DD II or III group, c—the significant difference between DD I and DD II or III group.

**Table 3:** CPB parameters—time of perfusion, aortic cross-clamp, and reperfusion.

Time (Min)	ND	DD I	DD II or III
Perfusion	63.42 (42–90)	61.45 (42–132)	56.3 (27–90)
Aortic cross-clamp	39.15 (24–70)	32.18 (19–52)	30.21 (15–50)
Reperfusion	20.57 (10–42)	22.72 (7–48)	17.79 (10–24)

Legend: CPB—cardiopulmonary bypass, ND—Normal LV Diastolic Function, DD I—Diastolic Dysfunction stage I, DD II or III—Diastolic dysfunction stage II or III.

**Table 4:** Postoperative data.

	ND	DD I	DD II or III	*p*
NA infusion, n (%)	9 (34.61)	3 (27.27)	6 (31.58)	NS
RBC transfusion, n (%)	5 (19.23)	2 (18.18)	5 (26.32)	NS
FFP transfusion, n (%)	4 (15.38)	1 (9.09)	2 (10.53)	NS
PLT transfusion, n (%)	4 (15.38)	1 (9.09)	2 (10.53)	NS
AF, n (%)	7 (26.92)	3 (27.27)	5 (26.32)	NS
PEEP max	6	6	6	NS
PEEP min	3	3	3	NS
Pressure—controlled ventilation n (%)	26 (100)	11 (100)	19 (100)	NS

Legend: ND—Normal LV Diastolic Function, DD I—Diastolic Dysfunction stage I, DD II or III—Diastolic dysfunction stage II or III, NA—Norepinephrine, RBC—Red Blood Cells, FFP—Fresh Frozen Plasma, PLT—Platelets, AF—Atrial Fibrillation, PEEP—positive end-expiratory pressure, NS—not significant.

**Table 5:** Primary endpoints.

Primary Endpoint	ND	DDI	DD II or III	*p*
Pneumonia	1 (3.85)	3 (27.27)	7 (38.89)	0.008 (a,b)
PaO2 min. MV	133.5 (109–148)	91 (83–131)	93.5 (82–112)	<0.001 (a,b)
PaO2 max. MV	176.69 (±28.23)	155.45 (±36.51)	133.44 (±35.45)	<0.001 (b)
PaO2/FiO2 min	318.5 (233–383)	237 (187—302)	204.75 (171–280)	0.001 (b)
PaO2/FiO2 max	425.5 (370–497)	353 (260–470)	262.5 (252–364)	<0.001 (b)
MV duration (h)	6.3 (5–8.3)	10.5 (7.55–16)	8.47 (6.15–10.4)	0.004 (a)

Legend: ND—Normal LV Diastolic Function, DD I—Diastolic Dysfunction stage I, DD II or III—Diastolic dysfunction stage II or III, PaO2 min. MV—minimal PaO2 during mechanical ventilation, PaO2 max.—the maximal PaO2 during mechanical ventilation; PaO2/FiO2 min—the lowest Horowitz index; PaO2/FiO2 max—the highest Horowitz index, a—the difference between ND and DD I group, b—the difference between ND and DD II or III group.

**Table 6:** Duration of mechanical ventilation, ICU stay length, and length of hospitalization and PaO2/FiO2 index in groups with and without pneumonia diagnosis.

Pneumonia Diagnosis	YES	NO	*p*
MV duration (h)	10.35 (7.48–10.75)	7.38 (5.35–0.03)	0.004
ICU stay length (h)	66 (56–83)	48 (36–57)	0.004
Hospitalization length (days)	8 (7–11.5)	6 (7–8)	0.045
PaO2/FiO2 index	296.5 (203–329)	243.5 (196–345)	0.85

Legend: MV—mechanical Ventilation. ICU—Intensive Care Unit, PaO2/FiO2 index—arterial blood partial pressure of oxygen to the fraction of inspired oxygen index.

**Table 7:** Secondary endpoints.

Secondary Endpoint	ND	DD I	DD II or III	*p*
ARDS, n (%)	0	0	0	NA
TRALI, n (%)	0	0	0	NA
ICU stay length (h)	46 (36–54)	51 (40–60)	64 (38–80)	NS
Hospitalization length (days)	7 (6–8)	8 (6–10)	7 (5–9)	NS
In-hospital mortality	0	0	0	NS

Legend: ND—Normal LV Diastolic Function, DD I—Diastolic Dysfunction stage I, DD II or III—Diastolic dysfunction stage II or III, ARDS—Acute Respiratory Distress Syndrome, TRALI—Transfusion—Related Acute Lung Injury, ICU—Intensive Care Unit, NA—not applicable; NS—not significant.

**Figure A1:**
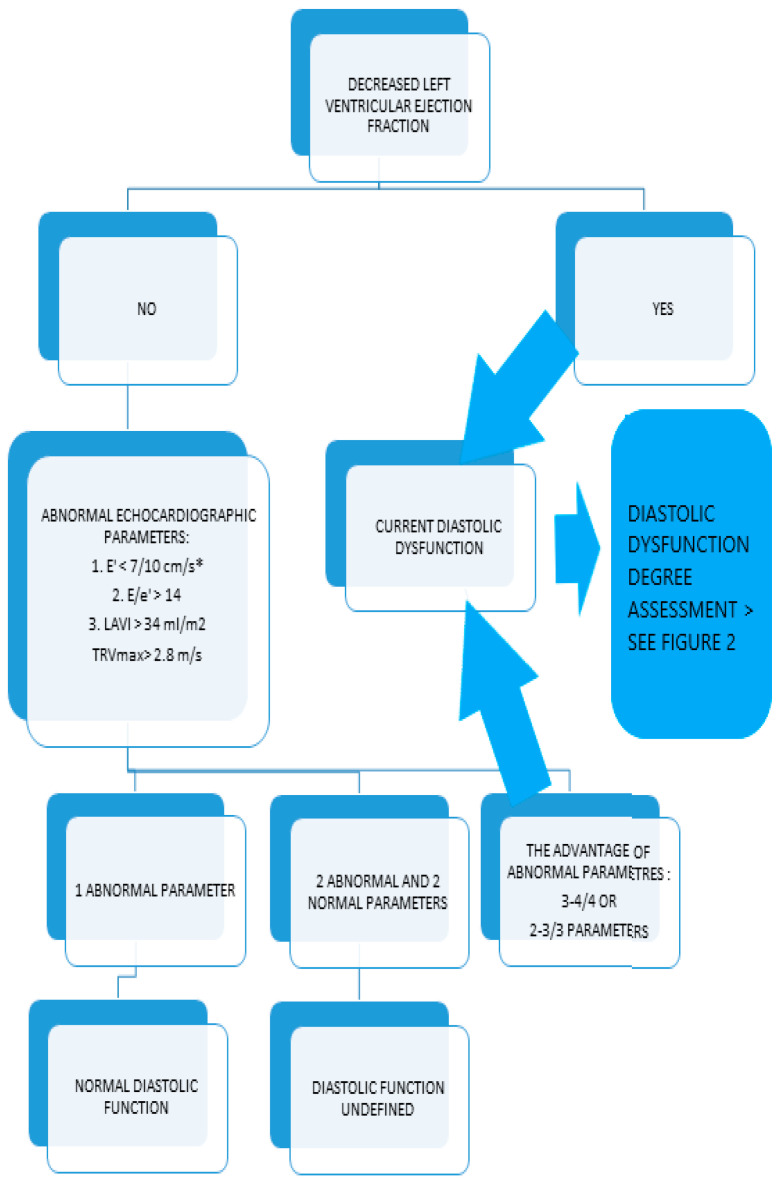
Echocardiographic assessment of left ventricular diastolic dysfunction—step 1: E—early mitral inflow velocity; e’—early diastolic mitral annular velocity; LAVI—left atrial volume indexed to body surface area; TRV—tricuspid valve regurgitation maximal velocity’; * 7 cm/s for septal early diastolic mitral annular velocity; 10 cm/s for lateral early diastolic mitral annular velocity.

**Figure A2:**
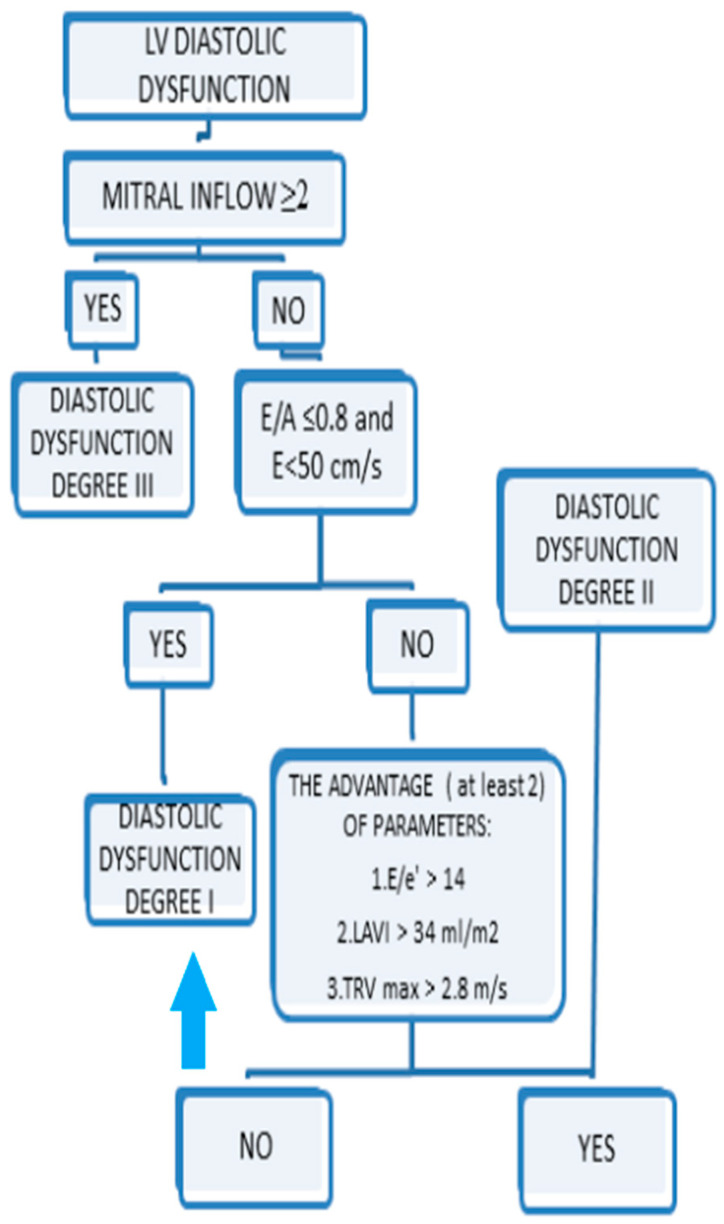
Echocardiographic assessment of left ventricular diastolic dysfunction—step 2: E—early mitral inflow velocity; A—late mitral inflow velocity (due to atrial contraction); e’—early diastolic mitral annular velocity; LAVI—left atrial volume indexed to body surface area; TRV—tricuspid valve regurgitation maximal velocity.

## Data Availability

The raw data will be available from the corresponding author upon a reasonable request.
